# A Functionalized Silicate Adsorbent and Exploration of Its Adsorption Mechanism

**DOI:** 10.3390/molecules25081820

**Published:** 2020-04-16

**Authors:** Hanzhi Lin, Tao Chen, Bo Yan, Zulv Huang, Yang Zhou, Jian Huang, Xianming Xiao

**Affiliations:** 1 State Key Laboratory of Organic Geochemistry, Guangdong Provincial Key Laboratory of Environmental Protection and Resources Utilization, Guangzhou Institute of Geochemistry, Chinese Academy of Sciences, Guangzhou 510640, China; xtulhz@126.com (H.L.); huangzulv18@mails.ucas.ac.cn (Z.H.); zhouyang2@gig.ac.cn (Y.Z.); xmxiao@gig.ac.cn (X.X.); 2 University of Chinese Academy of Sciences, Beijing 100082, China; 3 SCNU Environmental Research Institute, Guangdong Provincial Key Laboratory of Chemical Pollution and Environmental Safety & MOE Key Laboratory of Theoretical Chemistry of Environment, South China Normal University, Guangzhou 510006, China; bo.yan@m.scnu.edu.cn (B.Y.); 18371807641@163.com (J.H.); 4 School of Environment, South China Normal University, University Town, Guangzhou 510006, China

**Keywords:** calcination-activation, active silicate, heavy metal adsorption, adsorption mechanism

## Abstract

Active silicate materials have good adsorption and passivation effects on heavy metal pollutants. The experimental conditions for the preparation of active silicate heavy metal adsorbent (ASHMA) and the adsorption of Cu(II) by ASHMA were investigated. The optimum preparation conditions of ASHMA were as follows: 200 mesh quartz sand as the raw material, NaOH as an activating agent, NaOH/quartz sand = 0.45 (mass fraction), and calcination at 600 °C for 60 min. Under these conditions, the active silicon content of the adsorbent was 22.38% and the utilization efficiency of NaOH reached 89.11%. The adsorption mechanism of Cu(II) on the ASHMA was analyzed by the Langmuir and Freundlich isotherm models, which provided fits of 0.99 and 0.98, respectively. The separation coefficient (R_L_) and adsorption constant (n) showed that the adsorbent favored the adsorption of Cu(II), and the maximum adsorption capacity (Q_max_) estimated by the Langmuir isotherm was higher than that of 300 mg/L. Furthermore, adsorption by ASHMA was a relatively rapid process, and adsorption equilibrium could be achieved in 1 min. The adsorbents were characterized by FT-IR and Raman spectroscopy. The results showed that the activating agent destroyed the crystal structure of the quartz sand under calcination, and formed Si-O-Na and Si-OH groups to realize activation. The experimental results revealed that the adsorption process involved the removal of Cu(II) by the formation of Si-O-Cu bonds on the surface of the adsorbent. The above results indicated that the adsorbent prepared from quartz sand had a good removal effect on Cu(II).

## 1. Introduction

With the rapid development of the economy and society in recent years, heavy metal pollution in waters and soils due to mining, smelting and combustion emissions is becoming an increasingly serious problem [1,2,3]. Heavy metal contaminants pose a serious threat to human health and the environment owing to their harmful peculiarities such as nonbiodegradability, persistent toxicity and bioenrichment. Heavy metals pollution has led to many incidents around the world, such as Minamata disease, bone pain disease, and blood lead poisoning incidents [4,5,6]. Once the pollution occurs, it will have a long-term impact unless the heavy metals are removed from the contaminated site.

The amorphous silica structure of active silicate materials makes their reactivity much stronger than that of crystalline silica and confers these materials with excellent adsorption properties for pollutants such as heavy metals [7,8,9]. Studies have shown that the silanol groups (Si-OH) in active silicate materials are the main determinants of surface chemistry [10] and can form covalent bonds and hydrogen bonds with some ions and molecules [11]. Heavy metals such as Cu(II), Cd(II), and Pb(II) in aqueous solution were found to be removed by forming surface adsorption complexes with surface silanol [12,13]. Our team used leaching residue from lead-zinc tailings with high silicon content as a raw material to prepare an active silicate heavy metal adsorbent, which showed a very high adsorption capacity for the heavy metals Cu, Pb, Cd and Zn. The saturated adsorption capacities were 3.40, 2.83, 0.66 and 0.62 mmol/g, respectively [14].

Besides, when active silicate materials are applied to the remediation of heavy metals in soil, extractable heavy metals can be transformed into forms with relatively stable valence [15,16], and the heavy metal stress and accumulation in plants can be reduced [17]. Moreover, the active silicates can be absorbed by plants as nutrient elements to form phytoliths, which play an important role in increasing grain yield and enhancing photosynthesis, pest resistance, lodging resistance, and so on [18,19].

At present, most of the active silicate materials are solid wastes, such as steel slag, fly ash, tailings and so on [20,21]. However, these solid wastes may pose potential harm to the environment because of their complex chemical composition. Quartz sand is an inexpensive mineral with a wide range of sources, and its silicon dioxide content is high, so it is a potential raw material for the preparation of active silicate materials without threatening the environment.

In this study, an active silicate heavy metal adsorbent (ASHMA) was prepared by calcination activation at high temperature, and its adsorption characteristics and adsorption mechanism for Cu(II) in aqueous solution were investigated. As copper is a common heavy metal, this paper used Cu(II) as a material treatment index to carry out adsorption experiments. With this aim in mind, the factors and optimal conditions for the preparation of ASHMAs from quartz sand were investigated by using the active silicon content in the adsorbent and the utilization efficiency of the activator as indices. Langmuir and Freundlich models were used to study the adsorption capacity and adsorption characteristics of the adsorbent. The preparation and adsorption mechanisms of the adsorbent were determined by Fourier transform infrared spectroscopy (FT-IR) and energy dispersive X-ray spectroscopy (EDS). The results of this study are expected to enable the preparation of a safe, efficient and low-cost active silicate heavy metal adsorbent from cheap and widely available raw materials and accumulate basic data for its application to heavy metal contaminated wastewater treatment.

## 2. Results and Discussion

### 2.1. Preparation of ASHMA

#### 2.1.1. Production of ASHMA

The effects of calcination temperature (T), ratio of NaOH to quartz sand (R), calcination time (t), quartz sand particle size (S) and impurities (I) on the preparation of the active silicate heavy metal adsorbents were studied by the controlled variable method.

**Figure 1:**
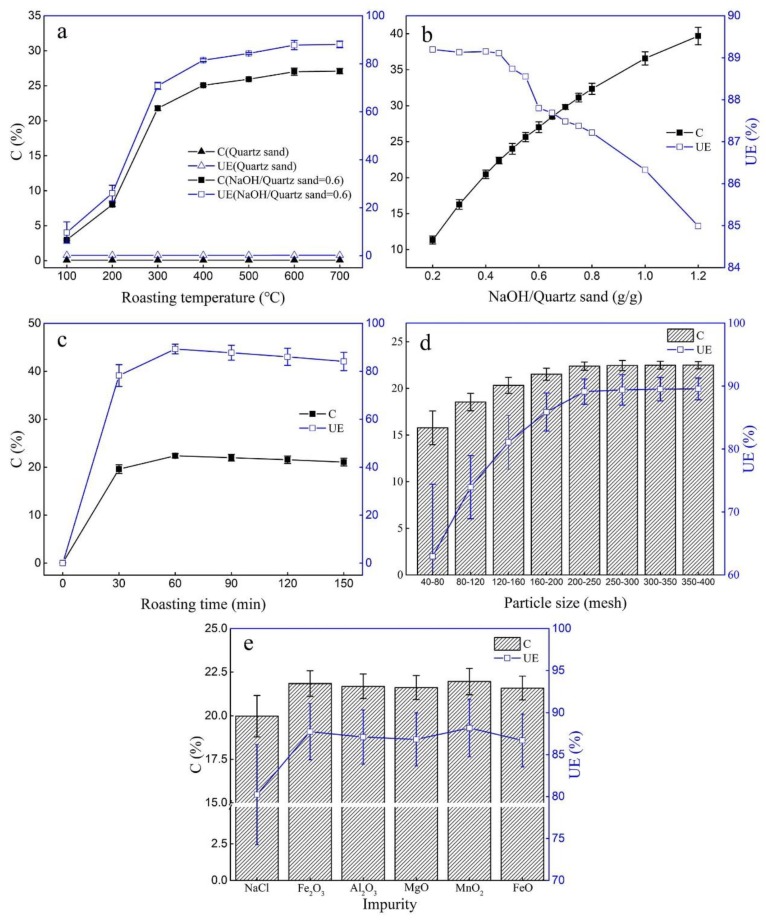
Effect of preparation conditions on active silicon contents. (**a**) roasting temperature ranged from 100 °C to 700 °C, (**b**) ratio of NaOH to quartz sand ranged from 0.2 to 1.2, (**c**) roasting time ranged from 0 min to 150 min, (**d**) practice size ranged from 40 mesh to 400 mesh and (**e**) impurities: NaCl, Fe2O3, Al2O3, MgO, MnO2 and FeO.

When the effect of temperature on the activation effect was investigated, the S was little than 200 mesh, R was 0 and 0.6, the calcination temperature increasing from 100 °C to 700 °C, and the calcination time was 60 min. It can be seen from Figure 1a that only increasing the temperature was insufficient to activate the quartz sand. After adding NaOH as an activator, the content of active silicon in the adsorbent increased with the increase of calcination temperature, and the content of active silicon became stable when the temperature exceeded 600 °C. Considering the activation efficiency and energy consumption, the optimum temperature for the preparation of ASHMA is 600 °C.

Figure 1b shows the effects of the ratio of NaOH to quartz sand (R), T was 600 °C, t was 60 min, and S was 200 mesh. With an increase in R from 0.2 to 1.2, the active silicon content increased from 11.34% to 39.69%. When the R was between 0.2 and 0.45, the UE remained stable. However, when the proportion increased from 0.45 to 1.2, UE decreased from 89.11% to 84.99%. Therefore, the optimum R was 0.45, and the content of active silicon in the adsorbent was 22.38% under these conditions.

For examining the effect of calcination time (t), T was 600 °C, t were ranged from 0 min to 150 min, the R was 0.45, the S was 200 mesh, the content of active silicon and UE increased rapidly with increasing t, and the activation effect was optimal when the t was 60 min (Figure 1c).

The effect of quartz sand particle size (S) is significant because a smaller particle size means a larger reaction area. It can be seen from Figure 1d that when the particle size was less than 200 mesh, UE increased rapidly with the decreasing of the particle size, while UE became stable when the particle size was greater than or equal to 200 mesh.

Many high-silica minerals have the potential for the preparation of silicate adsorbents, but it may contain a certain amount of impurities. The effects of different impurities (I) on the calcination activation process were investigated. The experiment was carried out under the condition that R was 0.45, T was 600 °C, t was 60 min, S was 200 mesh, and I was 1% of the mass of quartz sand. As shown in Figure 1e, the content of active silicon in the adsorbent decreased to varying degrees after the addition of impurities, and the effect was in the following order: NaCl > MgO > FeO > Al_2_O_3_ > Fe_2_O_3_ > MnO_2_. The content of available silicon was only 19.98% when adding 1% NaCl, while the content was 22.38% without impurities.

According to the above experiment, the optimum conditions for preparing the active silicate heavy metal adsorbent (ASHMA) were as follows, with T of 600 °C, R of 0.45, t of 60 min, S was lower than those were sifted with 200 mesh and no impurities added. The active silicon content of the adsorbents and the UE of NaOH under these conditions were 22.38% and 89.11%, respectively.

In order to investigate the influence of each factor on the preparation of ASHMA, the principal components analysis (PCA) of T, R, t, S and I were performed using the SPSS19.0 statistical package for Windows (IBM Corp, New York, NY, USA). The order of influence was as follows: particle size (S) > NaOH/quartz sand (R) > temperature (T) > impurity (I) > calcination time (t).

Through the component matrix, the factor model is expressed as Equation (1):

X = 0.915T + 0.969R + 0.692t + 0.971S + 0.927I(1)

#### 2.1.2. Characterization of ASHMA

To investigate the functional group changes in silicate materials before and after activation, Fourier transform infrared spectroscopy (FT-IR) was used to scan raw quartz sand (a), the ASHMA (b) was prepared with T of 600 °C, the t of 60 min, the S of 200 mesh and R of 0.45, ASHMA (c) was prepared with T of 600 °C, the t of 150 min, the S of 200 mesh and R of 0.45, ASHMA (d) was prepared with T of 600 °C, the t of 60 min, the S of 200 mesh, R of 0.45 and I of NaCl added. 

**Figure 2:**
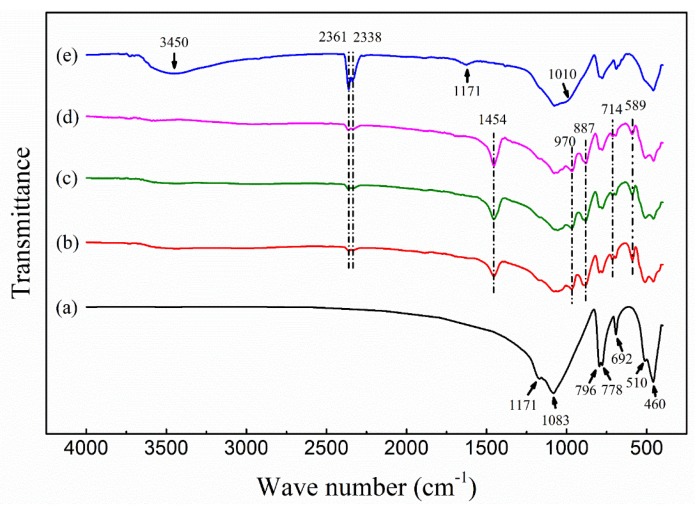
FT-IR spectra of quartz and ASHMAs.

As seen in Figure 2, the characteristic peaks of these three ASHMAs were basically the same. They all retained some characteristic peaks of quartz sand: the Si-O-Si anti-symmetric stretching vibration peak at 1171 cm^−1^ and the symmetrical stretching vibration peaks of Si-O at 510 cm^−1^, 692 cm^−1^, 778 cm^−1^, and 796 cm^−1^. The peak at 460 cm^−1^, which could be assigned to the deformation vibration of Si-O bonds [22,23] was almost constant, and its intensity can represent independent crystallinity [24]. Compared with the results for quartz sand, the peak intensity of these three adsorbents at 460 cm^−1^ decreased obviously, indicating that the independent crystallinity of the three adsorbents decreased. The order of intensity among the sample at 460 cm^−1^ was (b) < (c) < (d), which was exactly the opposite of their active silicon content ((b) > (c) > (d)), indicating that the strength of this peak can also indicate the degree of activation.

New absorption peaks of the adsorbents are observed at 589, 714, 887, 970, 1454, 2361 and 3450 cm^−1^. The newly developed Si-O stretching vibration peak at 589 cm^−1^ indicated that the SiO_2_ network had been damaged [25]. The wide peak of the adsorbent at 1000–1100 cm^−1^ was attributed to the breaking of the mesh structure of quartz during heating to form disordered SiO_4_ [14]. The tensile peak at 887 cm^−1^ was attributed to Si-O-Na [26]. The spectra also showed a Si-OH stretching peak at 970 cm^−1^ [27,28], and Si-OH and H-O-H stretching vibrational peaks at 1454 cm^−1^ and 3450 cm^−1^, respectively [29,30,31]. The results indicated that NaOH destroyed Si-O-Si bond under calcination, while Si-OH and Si-O-Na were formed at the same time to activate the silicon. In addition, the two weak peaks at 2338 cm^−1^ and 2361 cm^−1^ in the spectra were attributed to the stretching vibrations of CO_2_ [32,33], which was caused by the absorption of carbon dioxide from the air during the calcination process. Since there was no obvious difference in the properties of the adsorbents under different conditions, only ASHMA was further characterized.

**Figure 3:**
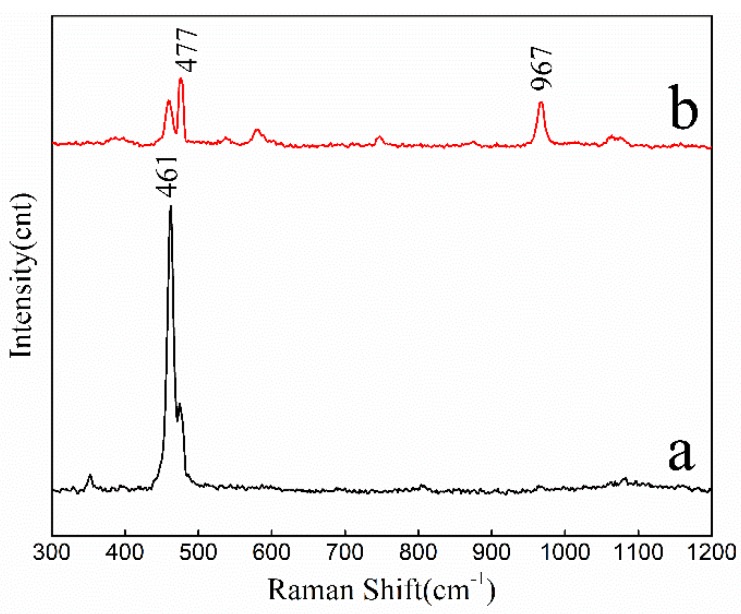
Raman spectra analysis of quartz sand (**a**) and ASHMA (**b**).

The Raman scanning spectra (Figure 3) showed that the peak intensity of the adsorbent at 461 cm^−1^ was distinctly weakened; this peak corresponds to the bending vibration of Si-O-Si silica tetrahedral bridge oxygen (Q^4^). The peak at 477 cm^−1^ corresponds to the internal deformation of silica tetrahedrons [34]. The peak at 967 cm^−1^ can be the overlap of the stretching vibrational peaks of two nonbridging oxygen silicone tetrahedron (Q^2^) and silane group Si-OH [35]. The characterization results of laser Raman analysis were consistent with those of FT-IR, the activator successfully destroyed the crystal structure of silica at high temperature, to realize activation.

**Figure 4:**
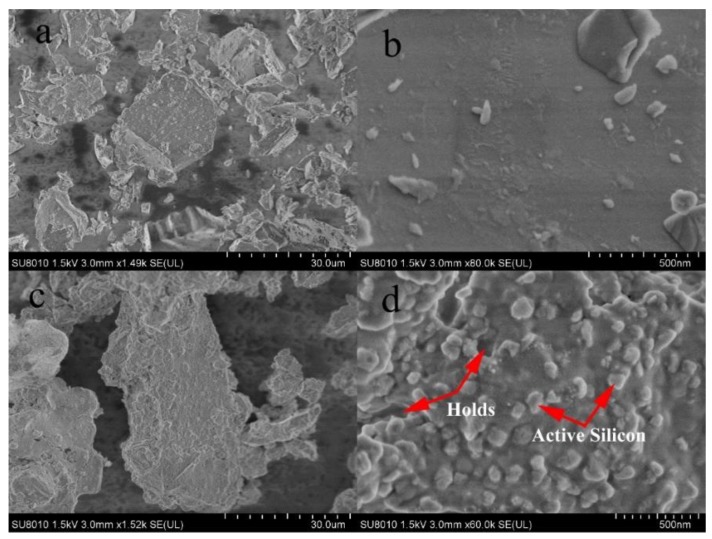
SEM images of quartz sand (**a**,**b**) and ASHMA (**c**,**d**), respectively.

The morphology of the adsorbent surface was observed by scanning electron microscopy. Quartz sand (Figure 4b) had a relatively flat surface and basically no pores. ASHMA (Figure 4d) developed some pores and formed a large number of 50–100 nm particles on the surface.

The surface of the absorbent articles shown in Figure 4d was analyzed by energy dispersive X-ray spectroscopy (EDS). The particles were mainly composed of Si, Na and O, and the mass concentration distribution showed good reproducibility. The results are shown in Table 1. According to the atomic ratio, it can be inferred that the chemical formula of the calcined product was Na_2_O·0.89SiO_2_·0.21H_2_O.

Considering that part of the NaOH is not reacted, the chemical formula of the product can be obtained by approximating the atomic ratio to Na_2_SiO_3_, and the utilization efficiency of NaOH was 88.65%. The result was in good agreement with 89.11% calculated by the formula (2). Therefore, it can be considered that the particles were active silicate components.

### 2.2. Adsorption

#### 2.2.1. Adsorption Properties

**Figure 5:**
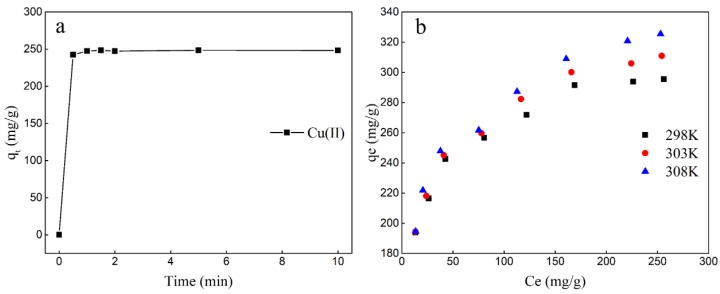
Effect of (**a**) equilibrium time on the adsorption of Cu(II) by the ASHMA and (**b**) temperature.

Figure 5a shows that the adsorption of Cu(II) by the active silicate heavy metal adsorbent was a rapid process, as 97% of the equilibrium adsorption capacity was reached in 0.5 min, and adsorption equilibrium was reached in 1 min. This may be attributed to the nanostructure of active silicate, which has a strong adsorption capacity for Cu(II). The experimental data fitted by the pseudo-second-order kinetics model (R^2^ = 0.992) was better than the pseudo-first-order kinetics model (R^2^ = 0.921) (Figure S2 and Table S1), suggesting that chemisorption was the leading factor in the uptake of Cu(II) by ASHMA. Meanwhile, the Q_e_ value calculated by pseudo-second-order kinetics is closer to those obtained by the experiment [36].

The effect of temperature on adsorption equilibrium was examined by adding 0.1–1.4 g/L adsorbent to 300 mg/L Cu(II) solution. As shown in Figure 5b, the equilibrium adsorption capacity increased with increasing temperature. Thermodynamic parameters such as standard enthalpy change (ΔH°), entropy change (ΔS°) and Gibbs free energy change (ΔG°) were estimated (Table S2). ΔH° values for ASHMA was 10.991 kJ/mol, the adsorption of Cu(II) was endothermic, which indicated that various binding mechanisms (such as ion exchange and chemical reactions) may be involved in the adsorption process [36,37]. Also, the ΔS° value calculated from the intercept was 89.143 J/(mol·K) for ASHMA. The positive value of ΔS° might be due to some structural changes between Cu(II) and the adsorbent during the adsorption process. The declining negative values of ΔG°, ranging from −15.574 kJ/mol to −16.465 kJ/mol with increased temperature from 298K to 308K, implied that higher temperatures are more thermodynamically favorable for this spontaneous feasibility of Cu(II) adsorption process [38].

**Figure 6:**
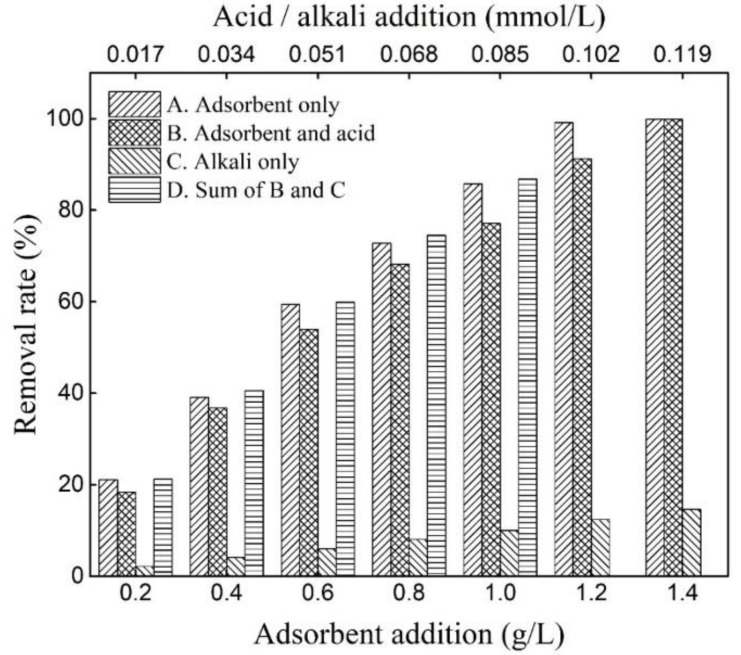
Effect of unreacted NaOH in the adsorbents on the adsorption efficiency of the adsorbents.

In the process of adsorbent preparation, the utilization efficiency of the activator (NaOH) was 89.11%. This result means that there was still unreacted NaOH in the adsorbent, which may affect adsorption. Therefore, the effect of unreacted NaOH in the adsorbent on the adsorption was investigated experimentally (Figure 6). Through experiments A and B, it can be seen that the adsorption effect of the adsorbent was weakened after the addition of nitric acid, indicating that the residual alkali in the adsorbent improved the adsorption performance by increasing the pH of the solution. Regardless of the amount of the ASHMA, the removal rate of experiment A was slightly lower than that of experiment D (the sum of experiments B and C), indicating that the excess alkali in the adsorbent had an insignificant inhibitory effect on the adsorption performance.

The recyclability of adsorption materials is a key index to evaluate the practical application potential of adsorption materials in wastewater treatment. Considering the obvious inhibition of adsorption performance at pH 2, a strongly acidic solution (0.2 M HCl) was used as eluent. The reusability of ASHMA for the adsorption of Cu(II) (dose = 1 g/L, C_0_ = 300 mg/L, t = 2 h, pH = 5 and T = 298 K) was investigated. As shown in Figure S5, the adsorption capacity decreased approximately 30.6% after a cycle, and the decline of that was more than 50.1% after five cycles.

#### 2.2.2. Adsorption Isotherm

In this research, the Langmuir and Freundlich models [39,40] were used to describe the equilibrium relationship between Cu (II) adsorbed by ASHMA and the remaining ions in aqueous solution. The Langmuir model assumes that the surface of the adsorbent is uniform and the adsorption occurs in a monolayer. The Langmuir model can be expressed by Equation (2):

(2) $$C_{e}/Q_{e}=1/K_{L}Q_{max}+C_{e}/Q_{max}$$

where K_L_ (L/mg) is a constant related to the adsorption energy and Q_max_ (mg/g) is the maximum adsorption amount of the adsorbent.

R_L_ is an important dimensionless parameter called the separation factor, which is determined by Equation (3):

(3) $$R_{L}=1/\left(1+K_{L}C_{ref}\right)$$

where C_ref_ is the equilibrium concentration of the solute in an arbitrary liquid phase. It is concluded that (i) 0 < R_L_ < 1 indicates favorable adsorption, (ii) R_L_ > 1 is unfavorable adsorption, (iii) R_L_ = 1 is linear adsorption, and (iv) R_L_ = 0 is irreversible adsorption.

The Freundlich isotherm is an empirical adsorption model based on nonuniform surface adsorption, and it can be expressed as Equation (4):

(4) $$lnQ_{e}=lnK_{F}+lnC_{e}/n$$

where K_f_ (mg/g) is the partition coefficient and n is a constant related to adsorption intensity; it is generally believed that n values between 1 and 10 correspond to easy adsorption.

**Figure 7:**
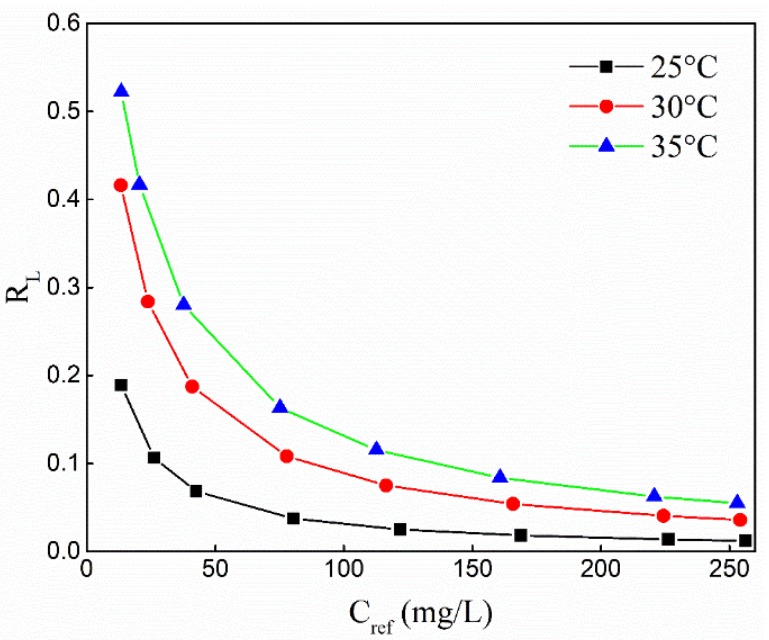
The relationship between the amount of ASHMA added and the separation coefficient R_L_ at different.

Table 2 summarizes the Langmuir and Freundlich model parameters for the adsorption of Cu(II) by the adsorbents. The adsorption properties of adsorbents fit well with the Langmuir model, and the correlation coefficient R^2^ was greater than 0.99, and the theoretical maximum adsorption capacity Q_max_ > 300 mg/g. Similarly, the Freundlich equation also matched the adsorption characteristics of Cu(II) well, and the correlation coefficient R^2^ was in excess of 0.97. The relationship between different amounts of adsorbent and separation coefficient R_L_ is shown in Figure 7.

Regardless of the differences in the doses added and changes in temperature, the value of R_L_ was always between 0 and 1, indicating that the adsorption of Cu(II) was favored [41]. The adsorption intensity constant n was in the range of 6.0 to 6.95, indicating that the adsorbent also favors the adsorption of Cu(II).

#### 2.2.3. Comparison with Other Adsorbents

Table 3 contrasts the adsorption capacity of some other silicate adsorption materials for copper ions in solution, such as vermiculite, kaolinite, zeolite, montmorillonite and mesoporous silica. Although the silicate content of these materials is high, their active silicon content is inferior, so their adsorption capacity is relatively limited, indicating that the activated silicon produced by calcination can significantly improve the adsorption capacity of silicate adsorbents for Cu(II).

#### 2.2.4. Adsorption Mechanism

According to the above model analysis, ASHMA had a good adsorption effect on Cu(II). To explore the adsorption mechanism of the adsorbents for Cu(II), FT-IR was used to characterize the adsorbents after adsorption at Ph = 5.0 ± 0.1 (Figure 2e). On the one hand, the peak of Si-O-Na at 887 cm^−1^ and the peak of Si-OH at 970 cm^−1^ disappeared after the adsorption of Cu(II). On the other hand, the enhanced absorption band at 1010 cm^−1^ was observed [45], which was considered to be a characteristic peak of a siloxy−copper bond (Si-O-Cu) structure. Furthermore, Cu(OH)_2_ and CuSiO_3_ precipitates could not be formed under the condition of pH 5 (Figure S3), which was confirmed by XRD characterization (Figure S1). Therefore, it can be considered that Cu(II) ions were adsorbed by the adsorbent, and the reaction that may occur in the adsorption process might be as follows:

2≡Si-ONa + Cu^2+^ → (≡Si-O)_2_Cu + 2Na^+^ (5)

≡Si-ONa + Cu(OH)^+^ → ≡Si-OCuOH + Na^+^ (6)

2≡Si-OH + Cu^2+^ → (≡Si-O)_2_Cu+ 2H ^+^ (7)

≡Si-OH + Cu^2+^ → ≡Si-OCu^+^ + H ^+^ (8)

≡Si-OH + Cu(OH)^+^ → ≡Si-OCuOH + H ^+^ (9)

The characteristic peak at 1625 cm^−1^ belonged to H-O-H bending vibration peak [41], and the tensile vibration peaks of -OH group and H_2_O at 3450 cm^−1^ were obviously strengthened, which might also be related to the existence of bound water.

**Figure 8:**
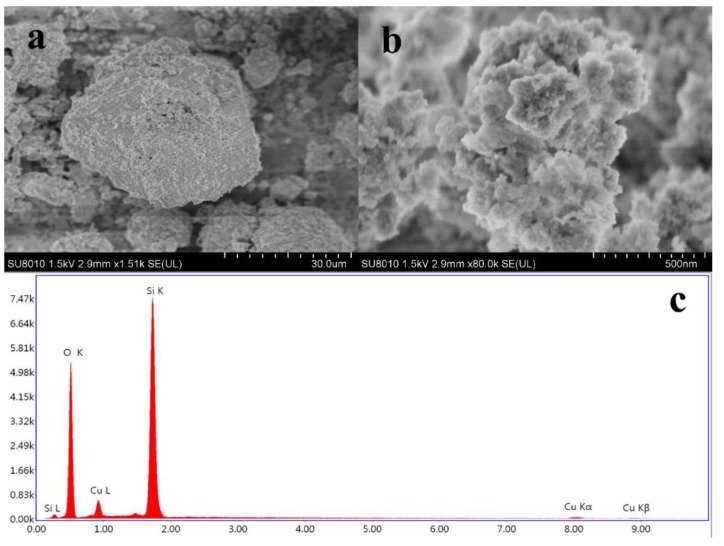
Scanning electron micrograph (**a**,**b**) and EDS pattern (**c**) for the ASHMA after the adsorption of Cu(II).

From the SEM results (Figure 8a), the surface morphology of ASHMA after adsorption of Cu(II) can be seen more directly. The surface of the adsorbent had a multilayer petal shape (Figure 8b). Combining the results of EDS spectrum analysis (Figure 8c), it was determined that the surface consisted of Si-O-Cu compound.

## 3. Materials and Methods

### 3.1. Materials

Quartz sand (purity ≥ 99.5%) was purchased from Guangzhou Chemical Reagent Factory (Guangzhou, China). The sand was crushed by an electromagnetic pulverizer, screened with a nylon screen into different particle size ranges, and loaded into sample bags for storage. Sodium hydroxide, sodium chloride, alumina, iron oxide, manganese dioxide, magnesium oxide, ferrous oxide and copper sulfate (CuSO_4_·5H_2_O) in the experiments were of analytical grade.

### 3.2. Preparation Process

The batch tests were carried out in a preheated muffle furnace. The quartz sand was mixed with the activator, then added to the crucible and roasted at a set temperature for a required time. The calcined samples were air-cooled, ground until completely homogenized, and passed through 60-mesh sieves for analysis.

The content of active silicon in the sample was extracted with 0.025 mol/L citric acid, and the content was determined by silicon molybdenum blue spectrophotometry according to the agricultural industry standard of the People’s Republic of China (Method for determination of soil available silicon, NY/T 1121.15-2006).

According to the requirements of the Chinese agricultural professional standard for silicate fertilizer (Silicon Fertilizer, NY/T 797-2004), as an active silicon material, the content of available SiO_2_ in ASHMA should be more than 20%, but the available silicon content in quartz sand is only 0.08%. To convert crystalline silicon into available silicon, quartz sand was mixed with sodium hydroxide and calcined at high temperatures. The reaction can be briefly described by reaction (10).

(10) $${\mathrm{SiO}}_{2}+2\mathrm{NaOH}≜{\mathrm{Na}}_{2}{\mathrm{SiO}}_{3}+H_{2}O$$

The utilization efficiency (UE) of the activator (NaOH) was calculated by determining the content of active silicon in the adsorbent, was calculated from Equation (11):

(11) $$\mathrm{UE}=\frac{n}{m_{NaOH}/M_{NaOH}}=\frac{2\times C\times m/M_{SiO_{2}}}{m_{NaOH}/M_{NaOH}}$$

where n (mol) is the molar number of NaOH; m_NaOH_ (g) is the added mass of NaOH; M_NaOH_ (g/mol) is the molar mass of NaOH; C (%) is the percentage of active silicon in ASHMA; m (g) is the mass of the calcined sample; and M_SiO_2__ (g/mol) is the molar mass of SiO_2_.

### 3.3. Adsorption Equilibrium

In this study, the adsorption experiments were carried out by adding 300 mg/L Cu(II) solution 100 ml to 250 ml stoppered Erlenmeyer flask and then adding other reagents. In the experiment for the batch adsorption of Cu(II), different masses of adsorbent (0.01–0.14 g) were added to the flask and the pH value was adjusted to 5.0 ± 0.1 with 1 mol/L nitric acid solution to avoid precipitation (Figure S3). The effect of residual NaOH on the adsorption effect of the adsorbent was investigated under the following operation conditions: A. Only adsorbent was added (0.02–0.14 g); B. Adsorbents with the same mass as that used in experiment A and nitric acid with the same molar number as the residual NaOH in the corresponding adsorbent were added; C. Only an amount of residual NaOH corresponding to the adsorption dose of experiment A was added to the solution. Then, the flasks were placed in a water bath oscillator for a certain time. After oscillation, the mixed solution was immediately centrifuged at 3000 rpm for 20 min, and the supernatant was filtered with 0.45 μm filters. Cu(II) concentration in the solution was determined by atomic absorption spectrophotometry (AAS, Hitachi ZA3000, Tokyo, Japan). The unit adsorption capacity of Cu(II) was calculated using Equation (12).

(12) $$Q_{e}=\left(C_{0}-C_{e}\right)V/m$$

where Q_e_ (mg/g) is the equilibrium adsorption capacity of the adsorbent, C_0_ (mg/L) is the initial concentration of the solution, C_e_ (mg/L) is the equilibrium concentration of the solution, m (g) is the amount of adsorbent, and V (L) is the volume of the solution. All experiments were repeated two times, and each point represents the average of repeated measurements.

### 3.4. Analytical Method

The content of active silicon in ASHMA was determined by an ultraviolet-visible spectrophotometer (PerkinElmer Lambda 850, Waltham, MA, USA) after color development. Functional group analysis was performed by Fourier transform infrared spectroscopy (FT-IR) (BRUKER VERTEX 70, Karlsruhe, Germany) in the wavenumber range of 400 cm^−1^ to 4000 cm^−1^ at room temperature, using the KBr disk transmission technique. The sample to KBr weight ratio was approximately 1:100. The functional groups were confirmed by a multifunctional automatic microscopic laser Raman spectrometer (HORIBA- JY Xplora, Paris, France), and scanning was performed at 532 nm with a fixed laser. The microscopic morphology and composition of the samples were observed and analyzed by scanning electron microscopy with energy dispersive X-ray spectroscopy (SEM, Hitachi SU8010; EDAX, OCTANESUPER, window area 60 square mm).

## 4. Conclusions

ASHMA was prepared by calcinating NaOH and quartz sand sifted to 200 mesh at a ratio of 0.45 at 600 °C for 60 min. Under these conditions, the content of active silicon in the adsorbent was 22.38%, and the utilization efficiency of the NaOH was 89.11%. It is estimated that the production cost of ASHMA is about 1000 RMB/t. Through the characterization of the structure and morphology of ASHMA, it was found that the Si-O-Si structure of quartz sand was destroyed and active functional groups such as Si-OH and Si-O-Na were formed. The adsorption of Cu(II) by ASHNA was an endothermic and rapid process, which was fitted by the Langmuir and Freundlich adsorption isotherm model. The values of the constant R_L_ and n indicated that adsorption was favorable, and the maximum adsorption capacity exceeded 300 mg/L when the adsorption temperature was 25 °C. According to the characterization of the precipitates after adsorption of Cu(II) by FT-IR and EDS, the Si-O-Cu structure was formed on the surface of the ASHMA. In conclusion, a safe, efficient and low-cost active silicate heavy metal adsorbent was prepared by a calcination-assisted method with quartz sand. ASHMA can be used to clean wastewater contained heavy metals and expected to be applied in the remediation of heavy metals contamination in soil.

## Figures and Tables

**Table 1:** Energy dispersive X-ray analysis of ASHMA.

	1	2	3	4	5	6	Mass Fraction (%)	Atomic Ratio	Approximation Ratio
O	43.74	41.41	42.43	40.13	42.52	41.64	41.98 ± 1.22	2.62	3
Na	35.71	39.03	38.10	36.79	36.15	40.30	37.68 ± 1.78	1.64	2
Si	20.55	19.56	19.47	23.08	21.33	18.06	20.34 ± 1.74	0.73	1

Note: mass fraction is the mean ± SD of the values from test 1 to 6; atomic ratio is the atomic molar ratio of O, Na and Si; approximation ratio is the approximate integer ratio of atomic ratio.

**Table 2:** Langmuir and Freundlich models for Cu(II) adsorption by ASHMA.

Temperature	Langmuir			Freundlich		
	Q_max_(mg/g)	K_L_(L/mg)	R^2^	K_f_(mg/g)	n	R^2^
298 K	303.95	0.321	0.9979	136.38	6.952	0.9791
303 K	326.80	0.106	0.9982	132.44	6.370	0.9864
308 K	338.98	0.068	0.9972	130.73	6.000	0.9802

**Table 3:** Comparison study of Cu(II) adsorption capacity.

Adsorbent Type	Initial Concentration of Cu(II) (mg/L)	Q_e_ (mg/g)	Dose (g/L)	References
Vermiculite	200	43.668	1.25	[42]
Kaolinite	250	10.1	2	[43]
Zeolite	100	14.3	20	[44]
Montmorillonite	200	27.6055	2	[45]
Mesoporous silica	100	142.932	2	[46]
This study	300	338.98	1.4	

## Supplementary Materials

The following are available online, Figure S1: XRD pattern of quartz sand, ASHMA(b) and ASHMA after adsorption of Cu(II), Figure S2: pseudo-first and -second-order kinetics of Cu(II) adsorption onto ASHMA (T = 298 K), Figure S3: Effect of pH on adsorption performance (with or without ASHMA), Figure S4: Adsorption effect of ASHMA on different concentrations of Cu(II) solution(Dose = 1 g/L, t = 2 h, pH = 5 and T = 298 K), Figure S5: The reusability of ASHMA for the adsorption of Cu(II) (Dose = 1 g/L, C0 = 300 mg/L, t = 2 h, pH = 5 and T = 298 K), Table S1: pseudo-first and second-order kinetics parameters of Cu(II) adsorption onto ASHMA (T = 298 K), Table S2: Values of thermodynamic parameters for the adsorption of Cu(II) onto ASHMA.
