# Supplementary material for: A Functionalized Silicate Adsorbent and Exploration of Its Adsorption Mechanism
Source: Molecules. 2020 Apr 16;25(8):1820. doi: 10.3390/molecules25081820 (PMC7221766; doi:10.3390/molecules25081820)
Supplement: Supplementary file 1 [file molecules-25-01820-s001.pdf]

Supplementary material

1. XRD pattern of quartz sand, ASHMA(b) and ASHMA after adsorption of Cu(II)

The quartz sand, ASHMA and ASHMA after adsorption of Cu(II) were characterized by an X-ray diffractometer (XRD, Rigaku MiniFlex 600, Japan) (Figure S1). The peak of Na_2SiO_3 was disappeared after adsorption of Cu(II), and no crystal phase of $Cu(OH)_2$ and $CuSiO_3$ were found. Compared with quartz sand, the intensity of the main peak of ASHMA decreased obviously, but no new crystal phase appeared, indicating that the adsorbed Cu(II) on the surface of ASHMA existed in an amorphous form. These results were consistent with the representation results of FT-IR and Raman.

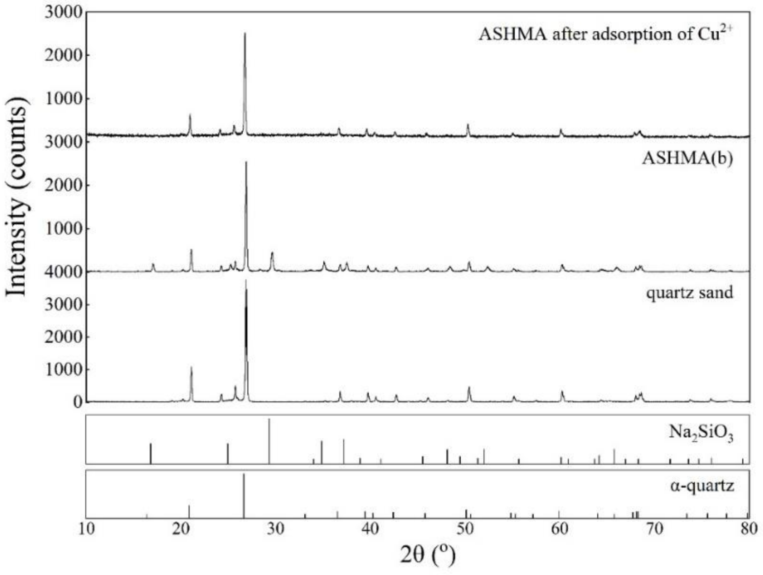

Figure S1. XRD pattern of quartz sand, ASHMA(b) and ASHMA after adsorption of Cu(II)

2. Pseudo-first-order and pseudo-second-order kinetic

The fit of the results of the experimental data to the pseudo-first-order model and pseudo-second-order model for ASHMA was carried out, according to Eq. (S2) and Eq. (S4), respectively. The calculations have been done for 285.57 mg/L Cu(II) solution initial concentration at 298K.

2.1 Pseudo-first-order kinetic equation

This well-known kinetic equation may be expressed as:

$$dQ_t/dt = k_1(Q_e - Q_t) \quad \text{(S1)}$$

where Q_e and Q_t are the amount of solute adsorbed per unit amount of adsorbent at equilibrium and any time, t, respectively (mg/g) and k_1 is the pseudo-first-order rate constant (min^{-1}). Integrating Eq. (S1) employing the boundary conditions that at t = 0, $Q_t = 0$, and that at t = t, $Q_t = Q_t$, the linear form of the equation becomes:

$$ln(Q_e - Q_t) = lnQ_e - k_1 t \quad \text{(S2)}$$

The adsorption rate constant, k_1 (min^{-1}), can be obtained from the slope of the linear plot of $ln(Q_e - Q_t)$ versus t.

2.2 Pseudo-second-order equation

The pseudo-second-order kinetic equation is:

$$dQ_t/dt = k_2(Q_e - Q_t)^2 \quad \text{(S3)}$$

where k_2 is the pseudo-second-order rate constant [g/(mg·min)]. On integration, employing the conditions that at t = 0, qt = 0 and that at t = t, qt = qt, this equation can be rearranged to give the linear

form:

$$t/Q_t = 1/k_2 \cdot Q_e^2 + t/Q_e \tag{S4}$$

The plot of t/Q_t versus t gives a linear relationship, which allows the values of Q_e and k_2 to be computed.

The kinetic parameters for all experimental data determined by pseudo-first-order and pseudo-second-order have been given in Table S1. The R^2 value of pseudo-second-order kinetics is 0.992, while that of pseudo-first-order kinetics is only 0.921, and the Q_e value calculated by pseudo-second-order kinetics is closer to that obtained by experiments than pseudo-first-order. It has shown that the mechanism concerning adsorption of Cu(II) on ASHMA can be explained by pseudo-second-order reaction kinetics.

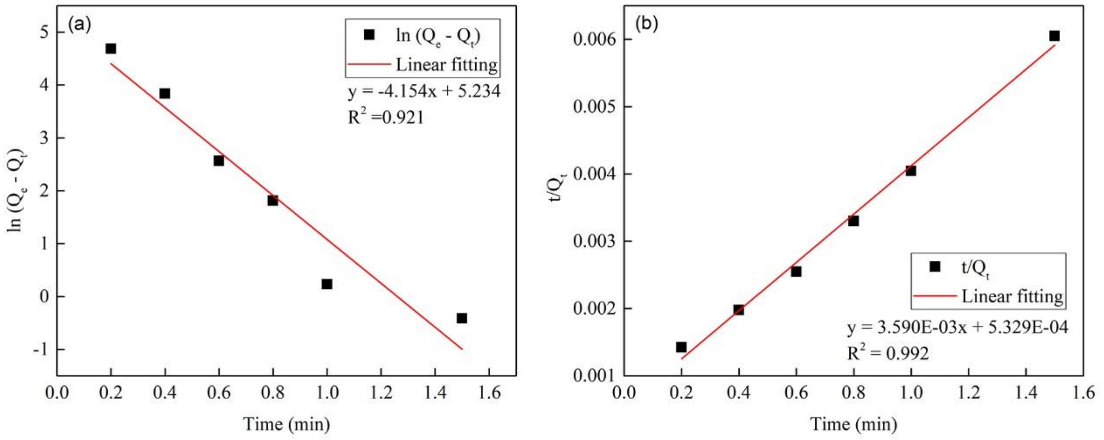

Figure S2. pseudo-first and -second-order kinetics of Cu(II) adsorption onto ASHMA (T = 298K).

Table S1. pseudo-first and-second-order kinetics parameters of Cu(II) adsorption onto ASHMA (T = 298K).

Initial Cu(II) concentration (mg/L)	Q_e, experimental (mg/g)	Pseudo-first-order			Pseudo-second-order		
		k_1 (min^{-1})	Q_e, calculated (mg/g)	R^2	k_2 [g/(mg ·min)]	Q_e, calculated (mg/g)	R^2
285.57	248.61	4.154	187.541	0.921	0.024	278.55	0.992

3. Thermodynamic parameters

The determination of the thermodynamic parameters of adsorption (ΔH°, ΔS°, and ΔG°) makes it possible to provide valuable information on the course of the reactions and the mechanisms that may exist. These parameters were determined for the temperature of 298, 303 and 308K.

The thermodynamic behavior of Cu(II) adsorption onto ASHMA was evaluated employing the following equations:

$$K_d = (C_i - C_e)/C_e \cdot V/m \tag{S5}$$

where C_i is the initial concentration (mg/L), C_e is the equilibration concentration after centrifugation (mg/L), V is the volume (mL) and m is the mass of diatomite (g), R [8.314 J/(mol ·K)] is the ideal gas constant, and T (K) is the temperature in Kelvin. ΔH °is the enthalpy change and ΔS is the entropy change in a given process. The values of enthalpy (ΔH °) and entropy (ΔS °) can be calculated from the slope and y-intercept of the plot of ln K_d versus 1/T via applying the equations.

Free energy changes (ΔG °) of specific adsorption are calculated from:

$$\triangle G^o = \triangle H^o - T \triangle S^o \tag{S6}$$

The constants of thermodynamics as shown in Table S2. ΔH ° values for ASHMA was determined

as 10.991 kJ/mol, successively. ΔH °value has a positive sign, it was realized that the adsorption of Cu(II) on ASHMA was endothermic. Also, the ΔS ° value calculated from the intercept was 89.143 J/(mol ·K) for ASHMA. The positive values of entropy may be due to some structural changes in the adsorbate and adsorbents during the adsorption process from aqueous solution onto the adsorbents. Besides, a positive value of ΔS ° indicates the increasing randomness at the solid-liquid interface during the adsorption of Cu(II) on the adsorbents. ΔG ° values are negative for Cu(II) adsorption on ASHMA and these values indicate that adsorption is spontaneous.

Table S2. Values of thermodynamic parameters for the adsorption of Cu(II) onto ASHMA.

C_i (mg/ L)	ΔH °(kJ/ mol)	Δ S °[J/(mol ·K)]	Δ G °(kJ/mol)		
			298K	303K	308K
285.57	10.991	89.143	-15.574	-16.019	-16.465

4. Effect of pH on adsorption performance (with or without ASHMA)

The pH effect experiment was carried out in the solution of Cu(II) with an initial concentration of 285.57 mg/L. The adsorption (or precipitation) effect of Cu(II) with ASHMA (1.4 g/L) and without ASHMA (0 g/L) at different pH values as shown in figure S3. The pH value was adjusted by 1 M nitric solution. Adding ASHMA had almost no adsorption effect on Cu (II) at pH 2. With the increase of pH value, the adsorption amount increased. Cu (II) concentration in the solution was 5.36 mg/L at pH 5 with ASHMA, and the removal rate could reach 98%, while the concentration of Cu (II) without ASHMA did not change. When pH > 5.2, the decrease of Cu(II) concentration might be caused by the double action of passivating agent and hydroxide precipitation.

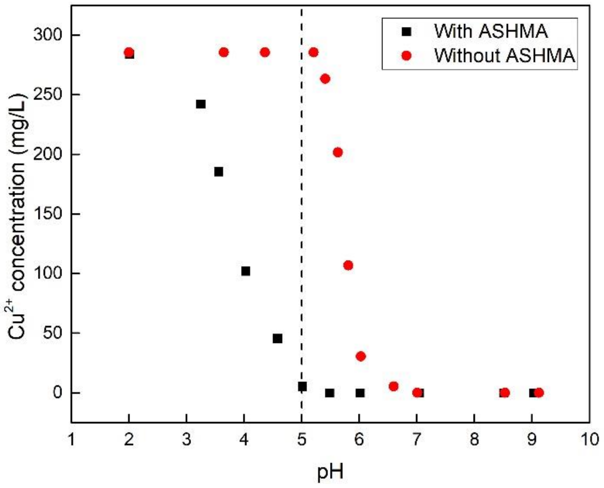

Figure S3. Effect of pH on adsorption performance (with or without ASHMA)

5. Adsorption effect of ASHMA on different concentrations of Cu(II) solution

Different concentration Cu(II) solution was tested, that the concentration of Cu(II) range from 5 mg/L to 500 mg/L (Dose =1 g/L, t = 2h, pH = 5 and T = 298K) (Figure S4). It can be found that the ASHMA has a good adsorption capacity for a low concentration of Cu(II), and the adsorption efficiency can even reach 100%.

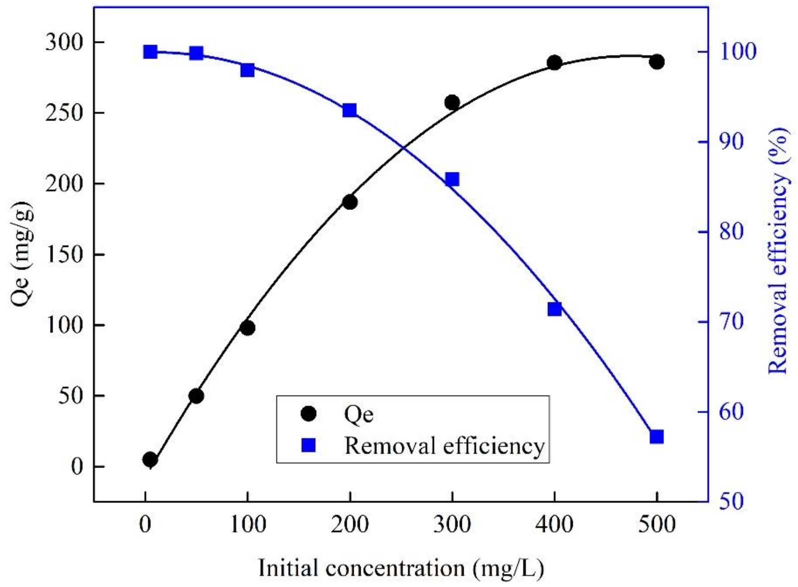

Figure S4. Adsorption effect of ASHMA on different concentrations of Cu(II) solution(Dose =1 g/L, t = 2h, pH = 5 and T = 298K).

6. The reusability of ASHMA for the adsorption of Cu(II)

Recyclability is the key performance to evaluate the practical application potential of adsorption materials in wastewater treatment. Considering the obvious inhibition of adsorption performance at pH 2, a strong acidic HCl solution (0.2 M) was selected as eluent. The reusability of ASHMA for the adsorption of Cu(II) (Dose =1 g/L, C_0 = 300 mg/L, t = 2h, pH = 5 and T = 298K). As shown in Figure S5, the adsorption capacity decreased by approximately 30.6% after a cycle, and while the decline of that was more than 50.1% in the five cycles. The performance of ASHMA recycling was poor.

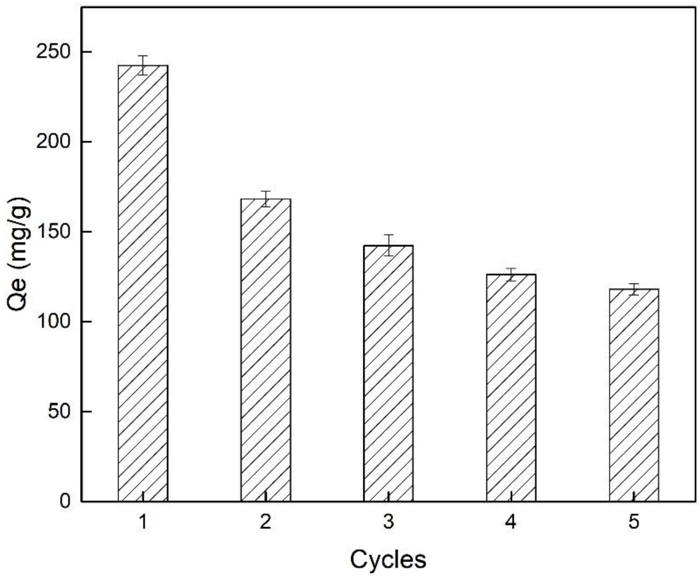

Figure S5. The reusability of ASHMA for the adsorption of Cu(II) (Dose = 1 g/L, C_0 = 300 mg/L, t = 2h, pH = 5 and T = 298K).
